# Annotations

**Published:** 1900-11-17

**Authors:** 


### ANNOTATIONS.

### The Domestic Uses of Electricity.

A SAD accident which occurred last week leads us to point out how little advantage is taken in our ordinary domestic arrangements of the facilities offered by modern invention. An elderly lady occupying a flat at Carlisle Mansions was burned to death. The household consisted of herself and her maid, and it was the custom of the latter, after settling her mistress in bed, to turn out the electric light and leave in the room some beef-tea and milk for use in the night, a candle and some matches being placed on the mantelshelf. The candlestick was out of reach and the deceased would have to get out of bed to get the matches. She was wearing a thin muslin night-dress. The maid had been in bed about half-an-hour when she heard her mistress scream, and, on going to her, found her one mass of flames.

The lady died, and that is the end of the sad story. But we think that there is a moral. Of course we do not impute blame in the slightest degree to anyone. What we are about to say is not generally thought of—it is in the mind of but few, and there is no neglect implied in not having had recourse to methods which are not yet in common use. Nevertheless, we cannot but feel distressed at the thought of an old lady living within reach of all kinds of modern inventions, and with electricity actually laid on into her bedroom, having to fumble about for matches, and night after night having to run the risk of being burned to death, a fate which ultimately overtook her; and we would urge that those who have to do with invalids and elderly people should make use to the full of the immense facilities offered by the electric current which is now laid on into so many houses. There is not the slightest difficulty in arranging for a person while lying in bed, even without putting the hands outside the bed-clothes, being enabled to procure a light, or ring for assistance, or turn on the current so as to warm the milk or beef tea placed by her bedside, or heat a footwarmer in the bed, or a radiator placed so as to take the chill off the air of the room, or to make it open or shut the window, or pull up the blind, or if required to light the gas-fire half-an-hour before getting up. Electricity is a little expensive, but to the wealthy it is a willing slave and can easily be made to do a host of things which elderly and weakly people especially require, and which, nevertheless, they seem especially to dislike asking others to do for them.

The simplest of all these things is the giving of light and the warming of food and footwarmers. It seems a satire on modern inventiveness that a lady, presumably in good circumstances, and with electricity already laid into her room, should be burned to death from fumbling with matches in the dark hours of the night.

### Meat Inspection.

WHEN English people go abroad they are apt to express unmitigated astonishment at the longsuffering patience with which foreigners put up with the worry of the octroi. That everybody entering within the town's boundaries should have to submit to an inspection, and that every parcel and basket should be liable to an inquisitorial search for taxable articles, seems to an ordinary go-as-you-please Englishman an intolerable interference with domestic liberty. There is, however, another side to all this. Directly it becomes a question of supervising a city's food supply, the fact that a point of inspection already exists by aid of which a complete watch can be kept over all food introduced from outside becomes a matter of great importance. In Paris, for example, there are no private slaughter houses, and all animals intended for human food must be killed at the public abattoirs, where the meat is examined by qualified inspectors acting under proper supervision, and if found satisfactory is marked with an official stamp. Of course, such a system would at once be rendered nugatory were meat admitted from outside. But such meat can only be brought in through the appointed toll-gates, and all meat so entering has at once to be taken either to the official slaughter houses or other appointed place where it can be inspected and stamped if found all right. In London we rather pride ourselves on being free from all such "absurd" restrictions, and seem to think it much more "English" to eat what we like. Who knows what we eat? The fact is that in regard to food, if as to nothing else, Londoners are like good little children in taking what is given them without a word. In Paris all is bad until it is stamped, while in London all is looked upon as good until it is found out to be the opposite—very much the opposite sometimes.

### The Rights of the Slum Proprietor.

THE complaint is general that when insanitary property is pulled down, and good healthy tenements put up in its place, the rents which must be charged for the new buildings are prohibitive for those who inhabited the old. One must qualify this statement by protesting that slummy people like slums, and will rather give the same rent for a place where they can be as dirty as they please than dwell in one where the proprietor insists on decent care being taken of the property. But nevertheless there is truth in the complaint. And if one asks why so high a rent is charged for decent accommodation the answer never is that the cost of building is high, but that it cost so much to purchase the property that had to be pulled down. It is often stated that when a city, or an improvement trust, or an association for building good dwellings for the poor, wants to buy a property, however disreputable and uninhabitable it may be, the price asked is one that would be large even for good property. Mr. William Tallack, of the Howard Association, has lately written to the *Times* protesting against the custom of giving the proprietors of slum property a fancy price when they are compelled to sell it, and Mr. Chamberlain recently stated that in some cases a specially high compensation had been paid to the owners of condemned property because it brought in a specially high rental through being let for immoral purposes! A writer in the *Daily News* says, "the compensation now payable by law to the owners of condemned areas is a monstrous fraud on the public." That is to say, men who ought to be punished are rewarded, and the community are forced to pay for an offence against themselves." Surely it is time the community saw to this. Perhaps a false tenderness plays the slum proprietor's game in many cases. Tenants are not evicted because they have nowhere to go. Property is not condemned out of sympathy for the tenants. Whatever right a proprietor may have to the value of his site, the fact that it is occupied by insanitary property should be taken as lessening that value, whereas at present it may even add to it.

